# Annual Meeting of the Chicago Medical Society

**Published:** 1862-04

**Authors:** 


### Proceedings of Societies.

### ANNUAL MEETING OF THE CHICAGO MEDICAL SOCIETY.

The Regular Annual Meeting of this Society was held in Larmon's Block, on the evening of the 4th inst.

The following officers were elected for the ensuing year:

For President, S. Wickersham, M. D.

Vice President, G. Paoli, M. D.

Secretary and Treasurer, E. L. Holmes, M. D.

Delegates to the State Medical Society, Drs. Orrin Smith, Thomas Bevan, S. Wickersham, E. L. Holmes.

After the election of officers, the Society proceeded with the usual order of business.

Dr. N. S. Davis, called the attention of members to the prevalence of scarlet fever, in the South Division of the city, and related the chief characteristics of several cases. Some of them were of a highly malignant character, showing early and alarming depression of the nervous centres. In one family on Franklin street, south of Adams, out of seven children, six had been attacked, of whom two died, one on the third, and the other on the fourth day. The other four recovered. The residence of this family is in a wet and unhealthy part of the city.

Indeed, most of the cases he had observed occurred in that part of the South Division lying between Clark street and the south branch of the river.

Dr. Wickersham, also stated that he had met with an increased number of cases in the same section of the city, and a larger ratio of mortality than usual.

Dr. Ammerman, reported two cases of disease of the femur, for the relief of which he had performed amputation at the hip-joint. The first was a boy in the County Alms-house, who had been attacked three months previously with severe periostitis of the lower third of the femur. At the time he was visited by Dr. A., the lower half of the thigh was greatly enlarged, measuring about 20 inches in circumference, dense to the touch, with many large tortuous veins visible on the surface, and several fistulous openings, chiefly on the inner side, through which a large amount of pus was discharged daily. The limb was removed by amputation at the hip; but the patient was too much exhausted, and died in about four days. The femur, which was presented for the examination of the Society, had undergone extensive morbid changes. The lower third was spongy and partially destroyed. The condyles had become spontaneously separated from the shaft of the bone, and were displaced backward and upward; while from the lower third of the shaft, numerous spicula of new bone had bone been formed, projecting at various angles from one to two inches in length. The texture of the bone throughout its whole length appeared to have been diseased, as it was found softened, and very vascular where it was cut through near the trochanters. Although this was reported under the name of *necrosis*, would not the description of the swollen part of the limb, and the state of the bone, justify us in placing it in the list of osteoid cancers, or malignant disease of the bone? Its progress was unusually rapid, only three months having elapsed since the first commencement of the disease.

The second case was that of a soldier, whose thigh bone was injured by a musket or rifle ball early last summer. From an examination of the femur, which was presented to the Society, it was evident that the primary injury was followed by extensive inflammation, and finally, death of the whole thickness of the bone from about two inches above the condyles to the trochanters. A case of new bone, of considerable thickness, had completely and closely invested the old, except a few openings near the original seat of injury. The history of the case was not given very fully, but the principal facts stated, were, that several months after the injury, the patient was brought to his home, a few miles from this city.

Dr. Amerman, on visiting him, found him much emaciated, and suffering from diarrhœa, hectic, and so much purulant discharge from the fistulous openings in the limb, that it was thought prudent to defer any operative procedure until the general health could be improved. After a short period of palliative treatment, an operation was undertaken for the purpose of removing the diseased and dead portion of the femur. The outer part of the thigh was laid open, and with the trephine and gouge, the case of new bone laid open freely; but the dead shaft of bone within was found so closely invested as to be immovably fixed in its position. A section, two or three inches in length was removed, but it was found impossible to remove the whole, without exsecting three-fourths ot the entire femur. Consequently, the wound was closed up, and suitable dressings applied to the limb. The patient bore the operation well, and for a few weeks his general health improved satisfactorily. After this, the hectic, diarrhœa, and other bad symptoms again supervened; and the patient and his friends became anxious to have the whole limb removed. This was done by Dr. A., who amputated at the hip. The patient died, however, in one or two days after the operation. The chief peculiarity in this case was, the extent of the necrosis, the amount of new bone encasing the old, and the entire failure of the process of exfoliation or separation of the dead from the living structure.

After resolving to hold its future meetings on the first Friday evening of each month, the Society adjourned.

---

Death of M. Bretonneau.—This distinguished physician has just died at an advanced age, at Passy, near Paris, where he occupied a noble villa. M. Bretonneau's name is connected with some of the most valuable discoveries in medicine, and had risen to great eminence by his teachings and original turn of mind. The two leading men of the medicine and surgery of Paris respectively, M. Trousseau and M. Velpeau, were his pupils.—*London Lancet.*

